# Non-additive effects of ACVR2A in preeclampsia in a Philippine population

**DOI:** 10.1186/s12884-018-2152-z

**Published:** 2019-01-08

**Authors:** Melissa D. Amosco, Gloria R. Tavera, Van Anthony M. Villar, Justin Michael A. Naniong, Lara Marie G. David-Bustamante, Scott M. Williams, Pedro A. Jose, Cynthia P. Palmes-Saloma

**Affiliations:** 1 National Institute of Molecular Biology and Biotechnology, National Science Complex, University of the Philippines, Diliman, 1101 Quezon City, Philippines; 2 Department of Obstetrics and Gynecology, Philippine General Hospital - University of the Philippines, Taft Avenue, 1000 Manila, Philippines; 3 Department of Population and Quantitative Health Sciences, Case Western Reserve University, School of Medicine, Cleveland, OH 44106 USA; 4 Division of Renal Diseases & Hypertension, Department of Medicine, The George Washington University of School of Medicine & Health Sciences, Washington, DC, 20037 USA; 5 Department of Pharmacology and Physiology, The George Washington University of School of Medicine & Health Sciences, Washington, DC, 20037 USA; 6 Philippine Genome Center, National Science Complex, University of the Philippines, Diliman, 1101 Quezon City, Philippines

**Keywords:** Association study, Multifactor dimensionality reduction, Philippines, Preeclampsia, Single nucleotide polymorphism

## Abstract

**Background:**

Multiple interrelated pathways contribute to the pathogenesis of preeclampsia, and variants in susceptibility genes may play a role among Filipinos, an ethnically distinct group with high prevalence of the disease. The objective of this study was to examine the association between variants in maternal candidate genes and the development of preeclampsia in a Philippine population.

**Methods:**

A case-control study involving 29 single nucleotide polymorphisms (SNPs) in 21 candidate genes was conducted in 150 patients with preeclampsia (cases) and 175 women with uncomplicated normal pregnancies (controls). Genotyping for the *GRK4* and *DRD1* gene variants was carried out using the TaqMan Assay, and all other variants were assayed using the Sequenom MassARRAY Iplex Platform. PLINK was used for SNP association testing. Multilocus association analysis was performed using multifactor dimensionality reduction (MDR) analysis.

**Results:**

Among the clinical factors, older age (*P* <  1 × 10–4), higher BMI (*P* <  1 × 10–4), having a new partner (*P* = 0.006), and increased time interval from previous pregnancy (*P* = 0.018) associated with preeclampsia. The MDR algorithm identified the genetic variant *ACVR2A* rs1014064 as interacting with age and BMI in association with preeclampsia among Filipino women.

**Conclusions:**

The MDR algorithm identified an interaction between age, BMI and *ACVR2A* rs1014064, indicating that context among genetic variants and demographic/clinical factors may be crucial to understanding the pathogenesis of preeclampsia among Filipino women.

**Electronic supplementary material:**

The online version of this article (10.1186/s12884-018-2152-z) contains supplementary material, which is available to authorized users.

## Background

Hypertensive disorders of pregnancy account for 36.7% of all maternal deaths in the Philippines [1], which is much higher than the worldwide rate of 18% [2]. Included among these hypertensive diseases affecting pregnant women is preeclampsia, a severe and diverse disorder that is associated with life-threatening multi-organ maternal complications and which causes serious feto-placental problems. It accounted for 22.5% of hypertensive patient admissions at the hospital where this study was conducted [3].

Preeclampsia is a multifactorial disease, with both genetic and environmental factors contributing to its development. Multiple interrelated pathways have been suggested to contribute to its pathogenesis. Previous studies have tested genes with potential biological relevance in specific pathways to ascertain whether certain variants influence the disease process. The biological pathways impacted by preeclampsia include but are not limited to aberrant placental development and dysfunctional hemodynamic and renal functions, impaired immune function, free radical dysregulation and lipid peroxidation, and defects in coagulation and fibrinolysis. We have previously found variants of the *VEGF-A* and *VEGFR1* genes to associate with preeclampsia among Filipinos, an ethnically distinct group with high prevalence [4]. These genes are important in angiogenesis, a critical process in the establishment of normal pregnancy and in preeclampsia.

The effect of single gene variation will likely be contingent on other genetic variations (gene-gene interaction, or epistasis) and environmental factors (gene-environment interaction). Since many genes and environmental factors interact to cause multifactor and polygenic diseases, including preeclampsia, the effect of any single gene may be too small to be detected using traditional statistical methods, which do not take these interactions into account**.** The multifactor dimensionality reduction (MDR) algorithm has been designed as an alternative to traditional statistical methods to deal with high-order gene/factor interactions [5, 6]. MDR has many other advantages over traditional methods. It is model-free, i.e., it does not assume any particular genetic model, and requires only a small sample size that can be used for case-control studies [5–7]**.** MDR has been successfully applied in detecting gene-gene interactions for a number of clinical phenotypes, which include bronchial asthma [8], autism [9], essential hypertension [10, 11], and type II diabetes [12].

In this study, we evaluated the role of contextual effects in the development of preeclampsia among Filipino women by analyzing 29 previously reported susceptibility SNPs found in 21 genes. The 21 genes are involved in various pathways that regulate the processes implicated in the development of preeclampsia and are as follows: endothelial/angiogenesis (*VEGFA, VEGFC, VEGFR1, VEGFR3*); dopaminergic system (*GRK4, DRD1*); renin-angiotensin system (*AGT*); immunity and inflammation (*ERAP2, CTLA4, IL1A, TNSF13B*); lipid metabolism (*LPL*); oxidative stress and detoxification (*eNOS, CYP1A2, PON1, EPHX1, GSTP1);* key signaling proteins (*ACVR2A*), hormone and neurotransmitter regulation (*COMT*) and protein biosynthesis (*MTR* and *MTRR*).

## Methods

This is a case-control study that included 381 individuals. Of these, 56 were removed in the final analysis because they had more than 2 genotypes missing. Of the 325 that remained, 150 were patients with preeclampsia (cases) and 175 were women with uncomplicated normal pregnancies (controls). Subjects were recruited upon admission to the hospital and were followed up until 6 weeks after delivery. Subjects included in the normal pregnancy control group had blood pressures ≤120/80 mmHg, consistent with the latest guidelines on hypertension [13]. Blood pressure was measured according to the Seventh Report of the Joint National Committee on Prevention, Detection, Evaluation, and Treatment of High Blood Pressure [14] and verified twice with at least a 4-h interval. Exclusion criteria for the control group were a history of hypertension and pregnancy-induced hypertension, multiple pregnancy, molar pregnancy, personal and family history of diabetes mellitus, ischemic heart disease, cerebrovascular accident, and renal disease.

Included in the preeclampsia group were patients who had a resting systolic blood pressure ≥ 140 mmHg and/or a diastolic blood pressure ≥ 90 mmHg and had proteinuria after 20 weeks of gestation. Proteinuria was defined as ≥300 mg protein in a 24-h urine collection or a urine protein dipstick of ≥2+. Exclusion criteria for the preeclampsia group were a history of hypertension, renal disease, proteinuria before the 20th week of pregnancy, multiple pregnancy, diabetes mellitus, ischemic heart disease, cerebrovascular accident, and renal disease.

This study was undertaken in accordance with the Declaration of Helsinki at the Department of Obstetrics and Gynecology of the University of the Philippines, Philippine General Hospital (UP-PGH) in Manila. All subjects provided informed written consent. The study was approved by the UP-PGH Ethics Review Board.

### SNP selection, blood sampling, DNA extraction, and genotyping

The candidate genes and respective variants were selected from different pathophysiological pathways involved in the development of preeclampsia and based on previous association studies. The *ACVR2A* SNPs in particular have been shown to be associated with preeclampsia in a study involving Brazilian [15] and Norwegian women [16]. Criteria for SNP selection included SNPs for which an association with preeclampsia has been reported in other ethnic populations and those that are also supported by the platform used for genotyping. The gene variants in relevant pathways include: endothelial/angiogenesis (*VEGFA* [17–19], *VEGFC* [20], *VEGFR1* [20–22]; renin-angiotensin system (*AGT* [23–25]); immunity and inflammation (*ERAP2* [26], *CTLA4* [27, 28], *IL1A* [29, 30], *TNSF13B* [31]); lipid metabolism (*LPL* [27, 32]); oxidative stress and detoxification (*eNOS* [33, 34], *CYP1A2* [35], *PON1* [36–39], *EPHX1* [40, 41], *GSTP1* [42])*;* key signaling proteins (*ACVR2A* [15, 16]), hormone and neurotransmitter regulation (*COMT* [43, 44]) and protein biosynthesis (*MTR* and *MTRR*) [45–48]. Also included are SNPs in the dopaminergic system (*GRK4* [49–52] and *DRD1* [52]), which have been established to be associated strongly with hypertension.

Venous blood samples for DNA extraction and genotyping were collected after a definitive diagnosis of preeclampsia or normal pregnancy. Three milliliters of venous blood were extracted at the time of hospital admission by venipuncture at the antecubital fossa, collected in a vacutainer with EDTA, and stored at 4 °C until DNA extraction. DNA was extracted from the peripheral blood mononuclear cells using the QIAamp® DNA Mini Kit. DNA purity and quantity were determined using a Nanodrop 2000 spectrometer (Thermo Scientific, Waltham, MA, USA). The DNA was stored at 4 °C until genetic profiling. Genotyping of the *GRK4* and *DRD1* variants was carried out using the TaqMan Assay at the University of Maryland Biopolymer-Genomics Core Facility. Genotyping of the other genes was carried out using the Sequenom MassARRAY Iplex Platform at the Center for Genomic Sciences of the University of Hong Kong. Repeat genotyping for 16 samples was performed for quality control.

### Statistical analysis

Statistical analysis for the clinical parameters was performed using STATA software, version 14.1 [53]. All values were expressed as mean/median ± standard error, while the association of known categorical risk factors was analyzed using Pearson’s chi-square test. Odds ratios (OR) were calculated to determine the odds of developing preeclampsia when the individual had the clinical factor of interest. OR was used for binary logistic regression and multinomial logistic regression. Minor allele frequencies and Hardy-Weinberg Equilibrium were calculated for each SNP using PLINK 1.9. Of the 29 SNPs, 6 were removed from further analysis (*AGT*, *LPL*, *FLT1, ERAP1, FLT4 and TNFSF13B*) because these SNPs were monomorphic, or had a minor allele frequency below 5%. Accumulated/average Cross Validation testing, training, consistency and permutation *P* values were calculated using MDR [6, 54]. The MDR algorithm and ViSEN software [55] were applied to the genetic data to enable the detection and characterization of epistatic SNP-SNP interactions and SNP-clinical factor (age and BMI) interaction. To identify a correct multi-locus model, the Acc. CV testing (Accumulated/average Cross Validation testing) and CV Consistency (Cross Validation Consistency) were calculated for each model.

## Results

Normal pregnancies and preeclampsia outcomes differed in several demographic and clinical characteristics. Mothers with normal pregnancies were significantly younger (25.2 vs. 30.3; *P* = < 1 × 10^− 4^) (Table 1) and had lower BMI (21.75 vs. 23.5; *P* = < 1 × 10^− 4^) (Table 1). The mean interval from last pregnancy was shorter in normal outcomes (2.03 years vs. 2.87 years; *P* = 0.018). Having a new partner also associated with preeclampsia (*P* = 0.006). However, neither smoking (*P* = 0.957) nor gravidity (*P* = 0.435) associated with preeclampsia (Table 2).

**Table 1:** Age, BMI, and interval year are risk factors for preeclampsia

Variable	Category	Mean/Median	*P* value
Age	NP	Mean: 25.2 ± 0.45 Median: 24	
	PE	Mean: 30.3 ± 0.53 Median: 31	< 1 × 10^−4^*
BMI^3^	NP	Mean: 21.75 ± 0.19 Median: 21.39	
	PE	Mean: 23.5 ± 0.21 Median: 23.31	< 1 × 10^−4^*
Interval Year	NP	Mean: 2.03 ± 0.21 Median: 1	
	PE	Mean: 2.87 ± 0.28 Median: 1	0.018*

Data are expressed as mean ± SEM and median; odds ratio (OR) used for binary logistic regression

*NP* Normal pregnancy, *PE* Preeclampsia, *BMI* Body mass index

*P values < 0.05 are statistically significant

The table summarizes the demographic, clinical characteristics, and risk factors of preeclampsia patients and controls with normal pregnancy. The preeclampsia patients were generally older, had higher BMI, and longer interval year from previous pregnancy compared with control subjects.

**Table 2:** A new partner is a risk factor for preeclampsia

Variable	Category	No	Yes	Pearson’s correlation	*P* value	OR
Smoking	NP	181	17			
	PE	167	16	0.003	0.957	1.020077
Alcohol	NP	184	14			
	PE	165	18	1.737	0.333	1.433766
Nulliparous	NP	109	89			
	PE	108	75	0.61	0.435	0.8504994
		Previous	New			
Partner	NP	173	25			
	PE^2^	140	43	7.67	0.006*	2.125

Data used odds ratio (OR) for binary logistic regression and for multinomial logistic regression

*NP* Normal pregnancy, *PE* Preeclampsia

*P values < 0.05 are statistically significant

Among the risk factors analyzed (smoking, alcoholic beverage consumption, nulliparity, and having a new partner) in preeclampsia patients and controls with normal pregnancy, only having a new partner was associated with increased risk of preeclampsia.

No SNPs associated with preeclampsia in the unadjusted analyses (Table 3). Association of single SNPs was also run adjusting for age, BMI, interval between pregnancies, and new partner (Additional file 1: Table S1). After adjusting for these covariates, one SNP reached nominal statistical significance (*VEGF-A* rs3025039; *P* = 0.022). This was not significant after adjusting for multiple testing (Bonferroni threshold *P* = 0.0023).

**Table 3:** Gene variants included in the study

Chr	SNP	Gene	Product (processes involved in)	MAF	OR	Allelic *P* value	Genotypic *P* value
1	rs1051740	*EPHX1*	Epoxide hydrolase (xenobiotic metabolism)	0.4547	1.039	0.8109	0.6148
1	rs699	*AGT*	Angiotensinogen (products elicit vasoconstriction)	0.1277	1.098	0.6899	NA
1	rs1805087	*MTR*	5-Methyltetrahydrofolate-Homocysteine Methyltransferase (methionine biosynthesis)	0.1357	1.013	0.9568	NA
2	rs3783550	*IL1A*	Interleukin 1-alpha (immunity, inflammation, hematopoiesis)	0.2031	1.003	0.988	0.7627
2	rs1014064	*ACVR2A*	Activin A Receptor Type 2A (growth and differentiation)	0.4414	0.8699	0.3805	0.556
2	rs2161983	*ACVR2A*		0.4385	0.8921	0.4717	0.5188
2	rs231775	*CTLA-4*	Cytotoxic T-Lymphocyte Associated Protein 4 (inhibition of immune responses)	0.4462	1.22	0.216	0.4626
4	rs2960306	*GRK4*	G protein-coupled receptor kinase 4 (receptor desensitization)	0.0679	1.763	0.0709	NA
4	rs1024323	*GRK4*		0.1354	1.26	0.3133	NA
4	rs1801058	*GRK4*		0.4599	1.024	0.8797	0.8192
4	rs7664413	*VEGF-C*	Vascular endothelial growth factor C (lymphangiogenesis)	0.2436	1.316	0.1412	0.181
5	rs1801394	*MTRR*	Methionine synthase reductase (methionine biosynthesis)	0.2754	0.9219	0.645	0.8572
5	rs2549782	*ERAP 2*	Endoplasmic reticulum aminopeptidase 2 (antigen processing)	0.4046	1.097	0.5622	0.8404
5	rs4532	*DRD1*	Dopamine D1 receptor (sodium transport, blood pressure regulation)	0.2105	1.01	0.9594	0.9148
6	rs2010963	*VEGF-A*	Vascular endothelial growth factor A (angiogenesis, vasculogenesis, endothelial cell growth & migration)	0.223	0.8585	0.4495	NA
6	rs3025039	*VEGF-A*		0.1188	0.6282	0.0626	NA
7	rs662	*PON1*	Paraoxonase 1 (inactivation of organophosphates, inhibition of atherosclerosis formation)	0.4306	0.9923	0.9612	0.9841
7	rs1799983	*NOS3*	Endothelial NOS (vascular relaxation, antioxidant activity)	0.1813	0.9324	0.7342	0.829
11	rs1695	*GSTP1*	Glutathione S-Transferase Pi 1 (xenobiotic metabolism)	0.2954	1.073	0.6809	0.8688
13	rs12584067	*VEGFR-1*	Vascular endothelial growth factor receptor 1 (cell proliferation and differentiation	0.0776	1.282	0.3978	NA
13	rs722503	*VEGFR-1*	Vascular endothelial growth factor receptor 1 (cell proliferation and differentiation	0.1660	0.8019	0.3593	NA
15	rs2470890	*CYP1A2*	Cytochrome P450 1A2 (xenobiotic metabolism)	0.0923	0.8182	0.4643	NA
22	rs4633	*COMT*	Catechol-O-methyltransferase (degrades catecholamines)	0.1692	1.056	0.7962	NA

*Chr* Chromosome, *SNP* Single nucleotide polymorphism, *MAF* Minor allele frequencies, *OR* Odds ratio, *NA* Not appplicable

The alleles and genotypes of the SNPs are not associated with preeclampsia when analyzed using PLINK**.** NA corresponds to the inability to determine a genotypic P value, due to a small number of counts in at least 1 of the 3 genotypes (i.e., AA/AT/TT not present, only AA/AT), thus, only allelic *P* value is given.

**Fig. 1:**
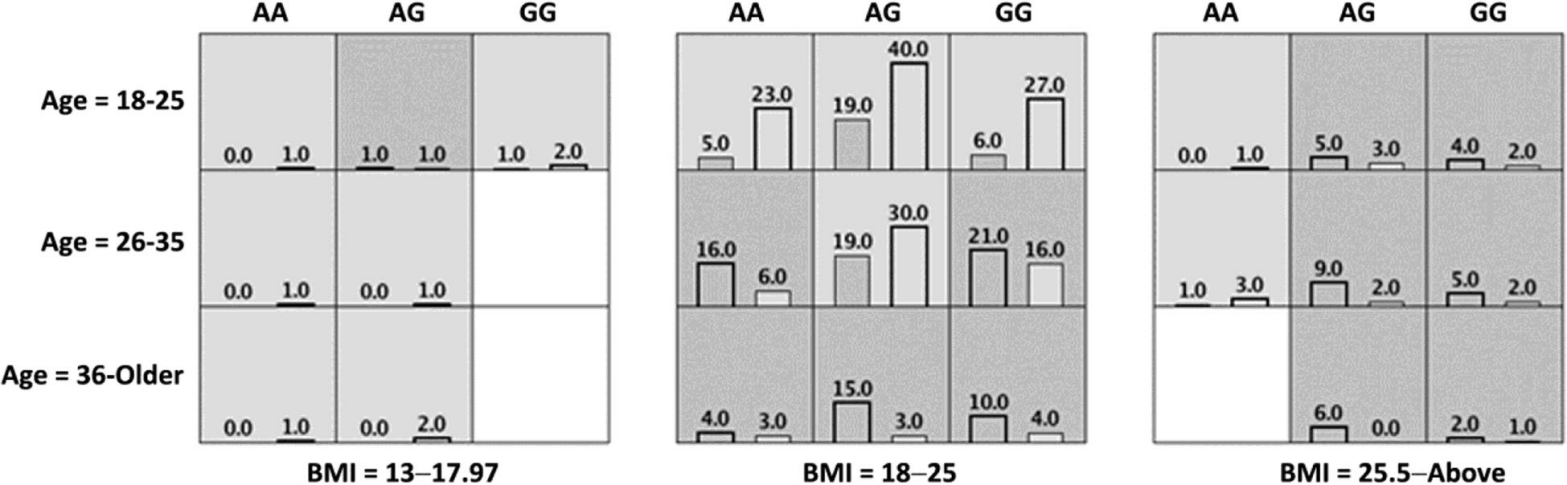
MDR model for interaction of rs1014064, age, and BMI. Each cell shows counts of preeclampsia on the left and normal pregnancy on the right. When re-analyzing only the middle range of BMI (18–25), genetics appeared to play a significant non-additive role in predicting preeclampsia, *P* < 1 × 10^− 4^, cross validation testing prediction = 64.88%, cross validation consistency = 10/10; MDR, multifactor dimensionality reduction; BMI, body mass index

Context-dependent effects were assessed using MDR. Only one model that included a genetic variant, as well as the significant demographic and clinical variables, was statistically significant; it included maternal age as three categories (18–25; 26–35; 36-Older); maternal BMI as three categories (13–17.97; 18–25; 25.1-Above) and rs1014064 (cross validation testing prediction = 60.95%; permutation *P* = 0.005 and cross validation consistency = 7/10) (Table 4). Upon examining the model, it was evident that the genetic effect was present primarily in the middle range of BMI (18–25) (Fig. 1). In low BMI ranges (13–17.97), the outcome was dominated by a protective effect and in high BMI ranges (25.1-above) by a risk-increasing effect of body mass. When re-analyzing only the middle range of BMI (18–25), genetics appeared to play a significant non-additive role (cross validation testing prediction = 64.88%; permutation *p* = < 1 × 10^− 4^ and cross validation consistency = 10/10; Fig. 1). There was no statistical epistasis detected using ViSEN.

**Table 4:** MDR analysis of genetic variants, adjusting for age and BMI

SNP rs1014064	GENE *ACVR2A*		
INTERACTION	Acc. CV Testing	CV Consistency	
Age	0.6457	10/10	
Age, BMI	06729	10/10	
rs1014064, Age, BMI	0.6095	7/10	*P* = 0.005*

There is an interaction between *ACVR2A* rs1014064, age, and BMI

*MDR* Multifactor dimensionality reduction, *SNP* Single nucleotide polymorphism, *BMI* Body mass index

*P value < 0.05 is statistically significant

The genetic data were analyzed for epistasis using MDR and ViSEN statistical software. Table 3 summarizes the different gene variants that were included in the analysis, as well as pertinent information for each, including the gene product and the processes in which it is involved, chromosome location, minor allele frequencies (MAF), and odds ratio (OR). Monomorphic SNPs (*VEGFR1* rs7335588, *AGT* rs41271499, and *LPL* rs268) and with MAF < 0.05 (*VEGFR3* rs307826, *ERAP2* rs17408150, and *TNFSF13B* rs16972194) were excluded from analyses. Genotype frequencies for all the SNPs were in Hardy-Weinberg equilibrium.

## Discussion

Non-linear interactions among multiple genetic and environmental or clinical factors are now understood to be important components in understanding the underlying pathogenesis, especially when considering the genetic bases of complex diseases such as preeclampsia. The MDR algorithm is a well-known data mining strategy that provides an improved representation of the genotypic and phenotypic data and enables better detection of higher-order interactions, such as epistatic interactions [6]. In the current study, MDR identified a four-locus model that underscores a possible interaction among rs1014064 (*ACVR2A*), rs7664413 (*VEGF-C*), rs2549782 (*ERAP2*), and rs662 (*PON1*) variants when un-adjusted for age or BMI (Additional file 2: Table S2). However, when age and BMI were adjusted for, these effects disappeared. A significant interaction was found in a model involving the genetic variant rs1014064 (*ACVR2A*) and the demographic and clinical variables, age and BMI.

The significant gene identified, *ACVR2A*, encodes the receptor for Activin A. ACVR2A expression in the placenta throughout pregnancy indicates its possible role in the regulation of placental development and function [56]. Initial studies have shown a linkage between preeclampsia and various parts of chromosome 2, where the *ACVR2A* gene is localized. The first reported locus for preeclampsia that met the criteria for genome-wide association significance was seen in chromosome 2p13 and 2q23 in a study involving Icelandic families, representing 343 affected women [57]. Two other genome-wide association studies identified other loci in chromosome 2 distinct from those seen in the initial study, i.e., 2p25 in 15 families with 49 affected women from Finland [58] and 2p11–12 and 2q22 involving 34 families, representing 121 affected women from Australia and New Zealand [59]. With the reported significant linkage to chromosome 2q22, the same group identified the *ACVR2A* gene as a strong positional candidate gene [60].

The MDR analysis identified the *ACVR2A* rs1014064, an intronic variant (A to G), as the only significant variant. This variant associated with preeclampsia in a large Norwegian population-based study (the HUNT study), together with the other *ACVR2A* variant (rs2161983) [16], which was also evaluated and found not associated with preeclampsia in this study, and with early onset preeclampsia in a Brazilian population [15].

MDR has been used to identify the important role of epistasis in polygenic disorders, such as sporadic breast cancer [6] and essential hypertension. A two-locus model including *ACE* and *GRK4* successfully predicted the blood pressure phenotype 70.5% of the time [10]**.** A genetic model based on the three common *GRK4* SNPs was 94.4% predictive of salt-sensitive hypertension, while a single-locus model with only the *GRK4* A142V variant was 78.4% predictive. By contrast, for low-renin hypertension, a two-locus model that includes the *GRK4* A142V variant and cytochrome P450 11B2 (*CYP11B2*) C-344 T was 77.8% predictive [11]. These results reflect the differences in the underlying genetics and the crucial role of epistasis in the development of the different hypertension-related phenotypes. Considering the spectrum of the clinical presentation of preeclampsia, it is conceivable that different phenotypes of the disease may involve specific gene polymorphisms, i.e., locus heterogeniety. In fact, the presence of severe forms of preeclampsia, HELLP syndrome and eclampsia, have been suggested to have their own set of predisposing gene variants. It is therefore important to know which specific genes contribute the most to their development.

We also used ViSEN software, which provides a global interaction map to identify and corroborate risk-associated SNPs, visualize putative gene interactions and generate an interaction or concept map for preeclampsia. Similar to what we observed using MDR, we detected no statistically significant two- and three-way epistatic interactions with ViSEN. Due to its limitation to analyze only up to three-way epistasis, the four-locus model that we observed with MDR was undetected. Moreover, ViSEN as a statistical tool has its own limitations, e.g., the statistical epistasis quantifications in ViSEN only consider discrete traits and cannot incorporate measures on continuous traits like age and BMI [55].

In the analysis of genetic datasets, an important consideration is the power of analytical methods to identify accurate predictive models of disease. The MDR approach overcomes the common setbacks found in other methods. It is non-parametric, model-free, and can identify high-order gene-gene interactions [6, 54, 61]. It retains its power to analyze in the presence of genotyping error and missing data (up to 5%). However, it has its own limitations, including a decrease in power in the presence of phenocopies and genetic heterogeneity [61].

With respect to marginal effects, one of the SNPs we genotyped in VEGF-A, rs3025039, that was previously identified as associating with preeclampsia in the Philippines [4] also showed a marginal association in our data set when adjusting for covariates (*p* = 0.022). Although this result would not stand after adjusting for multiple testing, as a replication it provided additional evidence that this variant confers pre-eclampsia risk especially since the direction of effect was the same in the present and the previous studies. A second SNP in this gene, rs722503, that was previously reported to be associated with preeclampsia, but only in pregnancies with women over 40, did not show evidence for significance in the current study (Additional file 1: Table S1). Of note, in the age and BMI adjusted model for rs722503 the *p* value did get smaller as compared to the unadjusted model, which is consistent with an age-related effect. This, however, was not surprising as very few pregnancies involved women over 40 (4). In addition, this SNP did not appear in our MDR analyses when all other SNPs were included.

A notable limitation of this study is the non-inclusion of other SNPs that have been shown to be associated with preeclampsia in specific ethnic groups, or more importantly in multi-gene meta-analysis studies involving different ethnic groups [25, 27, 62, 63] and in multigene association studies [64]. These include the gene variants *of FV, F2, ACE, SERPINE1, AGTR1, MTHFR,* and *MMP-9*. The *ACE* gene variant is an insertion/deletion polymorphism, which cannot be detected by the method used for genotyping in our studies. The other genes, although included in the initial list for analysis, were eventually dropped from the analysis due to technical problems. These genes, however, should be included in future studies.

## Conclusions

Preeclampsia is a multifactorial disease, with both genetic and environmental factors contributing to its development. Genetic variants from multiple, interrelated pathways have been suggested to contribute to the pathogenesis of the disease. The MDR algorithm enabled the analysis of high-order gene/factor interactions and identified *ACVR2A* rs1014064 as important in modulating preeclampsia risk among older Filipino women with a middle-range BMI.

## Additional files

Additional file 1: **Table S1.** Association of SNPs, adjusting for statistically significant risk factors. (DOCX 16 kb)

Additional file 2: **Table S2.** MDR analysis of genetic variants, without adjusting for age and BMI. (DOCX 14 kb)

## Abbreviations

*ACVR2A*: Activin A Receptor Type 2A
*AGT*: Angiotensinogen
*COMT*: Catechol-O-methyltransferase
*CTLA4*: Cytotoxic T-Lymphocyte Associated Protein 4
*CYP1A2*: Cytochrome P450 family 1 subfamily A member 2
*DRD1*: Dopamine D1 receptor
*eNOS*: Endothelial nitric oxide synthase
*EPHX1*: Epoxide hydrolase
*ERAP2*: Endoplasmic reticulum aminopeptidase 2
*GRK4*: G protein-coupled receptor kinase 4
*GSTP1*: Glutathione S-Transferase Pi 1
*IL1A*: Interleukin 1-alpha
*LPL*: *Lipoprotein lipase*
MDR: Multifactor dimensionality reduction
*MTR*: 5-Methyltetrahydrofolate-Homocysteine Methyltransferase
*MTRR*: Methionine synthase reductase
*PON1*: Paraoxonase 1
SNPs: Single nucleotide polymorphisms
*TNSF13B*: Tumor necrosis factor ligand superfamily member 13B
*VEGFA*: Vascular endothelial growth factor A
*VEGFC*: Vascular endothelial growth factor C
*VEGFR1*: Vascular endothelial growth factor receptor 1
*VEGFR3*: Vascular endothelial growth factor receptor 3
