# Supplementary material for: Non-additive effects of ACVR2A in preeclampsia in a Philippine population
Source: BMC Pregnancy Childbirth. 2019 Jan 8;19:11. doi: 10.1186/s12884-018-2152-z (PMC6323705; doi:10.1186/s12884-018-2152-z)
Supplement: Supplementary file 1 — Table S1. Association of SNPs, adjusting for statistically significant risk factors. (DOCX 16 kb) [file 12884_2018_2152_MOESM1_ESM.docx]

**TABLE S1 Association of SNPs, adjusting for statistically significant risk factors**

| **Chr** | **SNP** | **Gene** | **Product (processes involved in)** | **p** |
| --- | --- | --- | --- | --- |
| 1 | rs1051740 | *EPHX1* | Epoxide hydrolase (xenobiotic metabolism) | 0.66 |
| 1 | rs699 | *AGT* | Angiotensinogen (products elicit vasoconstriction) | 0.834 |
| 1 | rs1805087 | *MTR* | 5-Methyltetrahydrofolate-Homocysteine Methyltransferase (methionine biosynthesis) | 0.865 |
| 2 | rs3783550 | *IL1A* | Interleukin 1-alpha (immunity, inflammation, hematopoiesis) | 0.265 |
| 2 | rs1014064 | *ACVR2A* | Activin A Receptor Type 2A (growth and differentiation) | 0.954 |
| 2 | rs2161983 | *ACVR2A* |  | 0.862 |
| 2 | rs231775 | *CTLA-4* | Cytotoxic T-Lymphocyte Associated Protein 4 (inhibition of immune responses) | 0.356 |
| 4 | rs2960306 | *GRK4* | G protein-coupled receptor kinase 4 (receptor desensitization) | 0.154 |
| 4 | rs1024323 | *GRK4* |  | 0.215 |
| 4 | rs1801058 | *GRK4* |  | 0.87 |
| 4 | rs7664413 | *VEGF-C* | Vascular endothelial growth factor C (lymphangiogenesis) | 0.302 |
| 5 | rs1801394 | *MTRR* | Methionine synthase reductase (methionine biosynthesis) | 0.607 |
| 5 | rs2549782 | *ERAP 2* | Endoplasmic reticulum aminopeptidase 2 (antigen processing) | 0.598 |
| 5 | rs4532 | *DRD1* | Dopamine D1 receptor (sodium transport, blood pressure regulation) | 0.737 |
| 6 | rs2010963 | *VEGF-A* | Vascular endothelial growth factor A (angiogenesis, vasculogenesis, endothelial cell growth & migration) | 0.93 |
| 6 | rs3025039 | *VEGF-A* |  | **0.022** |
| 7 | rs662 | *PON1* | Paraoxonase 1 (inactivation of organophosphates, inhibition of atherosclerosis formation) | 0.75 |
| 7 | rs1799983 | *NOS3* | Endothelial NOS (vascular smooth muscle relaxation) | 0.958 |
| 11 | rs1695 | *GSTP1* | Glutathione S-Transferase Pi 1 (xenobiotic metabolism) | 0.203 |
| 13 | rs12584067 | *VEGFR-1* | Vascular endothelial growth factor receptor 1 (cell proliferation and differentiation | 0.313 |
| 13 | rs722503 | *VEGFR-1* |  | 0.115 |
| 15 | rs2470890 | *CYP1A2* | Cytochrome P450 1A2 (xenobiotic metabolism) | 0.326 |
| 22 | rs4633 | *COMT* | Catechol-O-methyltransferase (degrades catecholamine) | 0.585 |

*Chr*, chromosome; *SNP*, single nucleotide polymorphism

The association of SNPs was run adjusting for age, BMI, interval between pregnancies, and new partner in STATA. After adjusting for these covariates, one SNP reached statistical signficance (*VEGF-A* rs3025039; P = 0.022). This was not significant after adjusting for multiple testing (Bonferroni threshold P = 0.0023). Logistic regression includes all significant risk factors.
