# Supplementary material for: Non-additive effects of ACVR2A in preeclampsia in a Philippine population
Source: BMC Pregnancy Childbirth. 2019 Jan 8;19:11. doi: 10.1186/s12884-018-2152-z (PMC6323705; doi:10.1186/s12884-018-2152-z)
Supplement: Supplementary file 2 — Table S2. MDR analysis of genetic variants, without adjusting for age and BMI. (DOCX 14 kb) [file 12884_2018_2152_MOESM2_ESM.docx]

**TABLE S2**

**MDR analysis of genetic variants, without adjusting for age and BMI**

| **SNP** | **GENE** |  |  |  |  |
| --- | --- | --- | --- | --- | --- |
| rs1014064 | *ACVR2A* |  |  |  |  |
| rs7664413 | *VEGF-C* |  |  |  |  |
| rs2549782 | *ERAP 2* |  |  |  |  |
| rs662 | *PON1* |  |  |  |  |
| **INTERACTION** | | | **Acc. CV Testing** | **CV Consistency** |  |
| rs1014064, rs7664413, rs2549782, rs662 | | | 0.6281 | 9/10 | p = 0.001 |

*MDR*, multifactor dimensionality reduction; *SNP*, single nucleotide polymorphism; *BMI*, body mass index

There is an interaction among rs1014064 (*ACVR2A*), rs7664413 (*VEGF-C*), rs2549782 (*ERAP 2*), and rs662 (*PON1*), when unadjusted for age and BMI
